# An Analysis of *IL-10*, *IL-17A*, *IL-17RA*, *IL-23A* and *IL-23R* Expression and Their Correlation with Clinical Course in Patients with Psoriasis

**DOI:** 10.3390/jcm10245834

**Published:** 2021-12-13

**Authors:** Magdalena Kutwin, Monika Migdalska-Sęk, Ewa Brzeziańska-Lasota, Piotr Zelga, Anna Woźniacka

**Affiliations:** 1 Department of Dermatology and Venereology, Medical University of Lodz, 90-647 Lodz, Poland; anna.wozniacka@umed.lodz.pl; 2 Department of Biomedicine and Genetics, Medical University of Lodz, 92-213 Lodz, Poland; monika.migdalska-sek@umed.lodz.pl (M.M.-S.); ewa.brzezianska@umed.lodz.pl (E.B.-L.); 3 Department of Surgery, University of Cambridge, Cambridge CB2 0QQ, UK; piotr_zelga@op.pl

**Keywords:** cytokines, disease activity, immunology, molecular markers, psoriasis, skin disease

## Abstract

Being one of the most common dermatological inflammatory disorders, psoriasis is a frequent subject of research. It is considered to be a T cell-dependent immune disease whose pathogenesis is influenced by cytokines, such as *IL-10*, *IL-17A*, *IL-17RA*, *IL-23A* and *IL-23R*. The present study examines whether the expression of selected genes is correlated with the clinical course of psoriasis, assessed by the PASI, BSA and DLQI scales. Skin biopsies and blood from 60 patients with psoriasis and 24 healthy controls were obtained for RNA isolation. These were subjected to RT-PCR for *IL-10*, *IL-17A*, *IL-17RA*, *IL-23A* and *IL-23R* genes. The results were presented as an RQ value. *IL-17A* and *IL-23R* expression levels were higher in psoriatic skin compared to controls, while *IL-10* expression was lower. A positive correlation was also found between RQ for *IL-23A* and PASI index. Psoriatic skin is characterised by elevated expression of *IL-17A* and *IL-23R* and decreased expression of *IL-10*. This indicates that the selected cytokines may be one of the factors involved in the pathogenesis and pathomechanism of psoriasis, but more studies need to be made before we can elucidate the exact reason for the unbalance in cytokine expression levels.

## 1. Introduction

Psoriasis vulgaris is a chronic, recurrent dermatosis causing erythematosus and scaly skin plaques [1,2,3,4,5,6]. It is regarded as the most common T cell-mediated inflammatory disease in humans, with a mean prevalence in Caucasian populations of 2–3% [7,8,9,10,11,12,13]. Men and women are equally often affected [14,15]. It is an immunologically mediated disease, but genetic and epigenetic factors also play an important role in its development [16,17,18,19,20,21]; however, these may be modulated by initiating environmental factors, such as infections, medications, injuries, stress or alcohol abuse [19,20,22,23,24,25,26]. Psoriasis often coexists with other autoimmune diseases, such as rheumatoid arthritis, autoimmune thyroid diseases, inflammatory bowel diseases, celiac disease and vitiligo [27,28,29,30,31].

However, until now, the exact etiopathogenesis of psoriasis vulgaris and the mechanisms leading to its development remain unclear. Despite this, recent studies suggest that immunological mechanisms associated with Th1 and Th17 lymphocytes play a dominant role in the development of psoriatic lesions, with the activation of the interleukin (IL)-12/Th1/interferon (IFN)-γ and *IL-23*/Th17/*IL-17* pathways being critical. The stimulation of Th1 lymphocytes to secrete pro-inflammatory molecules, such as tumour necrosis factor (TNF)-α, IFN-γ, IL-2 and IL-3 leads to the induction and maintenance of skin inflammation and an increase in the number of keratinocytes in the epidermis. In turn, Th17 lymphocytes activated by *IL-23* begin to secrete effector cytokines, such as *IL-17*, TNF-α, *IL-22* or *IL-6*. These cytokines lead to the inflow of neutrophils and other inflammatory cells to the skin and epidermis, excessive proliferation of keratinocytes and parakeratosis [28,32,33,34,35,36,37,38].

*IL-10* is also believed to play a role in the pathogenesis of psoriasis by inhibiting the formation of antigen-induced Th1 lymphocytes, as well as the production of cytokines, particularly IFN-γ and IL-2, and the secretion of TNF, *IL-1α*, *IL-6*, *IL-8*, *IL-12*, granulocyte colony-stimulating-factor (G-CSF) and granulocyte macrophage colony-stimulating-factor (GM-CSF) by macrophages and monocytes. In addition, *IL-10* enhances the adaptive abilities of regulatory T cells. Together, these immunological processes result in the manifestation of clinical symptoms in the form of a dermal-epidermal papule covered with silvery-white scales [39,40].

The aim of the study is to determine the role of selected genes *viz. IL-10*, *IL-17A*, *IL-17RA* (interleukin 17A receptor), *IL-23A* (interleukin 23 subunit α) and *IL-23R* (interleukin 23 receptor) in the development of psoriasis. The study determined their expression (RQ—relative quantification) in regulatory T cells in blood and skin biopsies taken from patients with psoriasis, and correlated them with samples from healthy volunteers. All genetic parameters were compared with selected clinical indicators of the activity and severity of the disease process in patients: the PASI (Psoriasis Area and Severity Index), BSA (Body Surface Area) and DLQI (Dermatology Life Quality Index) ratios.

## 2. Materials and Methods

### 2.1. Clinical Features of Patients and Skin Samples

The research covered a group of 60 adults suffering from plaque psoriasis, 18 women (30%) and 42 men (70%), aged from 23 to 83 years (mean age 51.22 ± 15.95 years). The patients were treated in the Department of Dermatology and Venereology and/or in the Diagnostic and Treatment Centre of Skin Diseases between March 2016 and February 2020. After obtaining written consent from the patients, a 4 mm diameter sample was taken from the area of the skin lesion by punch-biopsy, and 10 mL of venous whole blood was collected into EDTA anticoagulant.

The control group was matched for gender and age. It comprised 24 healthy people (11 women and 13 men), aged from 29 to 79 years (mean age 51.42 ± 16.34 years), without family history of the presence of psoriasis. Skin biopsies were taken by a surgeon. Exclusion criteria from participation in the study included pregnancy, lactation and the use of drugs that excessively prolong bleeding time.

### 2.2. Isolation of T-regulatory Lymphocytes (CD4/CD25) from Whole Blood

From venous whole blood collected from all patients, T-regulatory (T-reg) lymphocytes were isolated. The principle of isolation of CD4/CD25 cells from blood is based on the protocol of two-step selection of T-regulatory lymphocytes (T-reg), according to Sugiyama H. et al., 2005 [41] (with minor modifications and the use of kits StemCell Technologies, Vancouver, BC, Kanada). Firstly, CD4+ cells were obtained by negative selection using the RosetteSep^®^Humane CD4+ T Cell Enrichment Cocktail kit (StemCell Technologies), according to the manufacturer’s instructions. Following this, CD25+ T lymphocytes were positively selected from enriched CD4+ cells using the EasySep^®^ Human CD25 Positive Selection Cocktail kit (StemCell Technologies) and magnetic beads EasySep^®^ Magnetic Nanoparticles (StemCell Technologies). The positively-selected cells were resuspended in 1 × PBS and used for RNA isolation.

### 2.3. Homogenisation of Skin Biopsies

Skin samples immediately after excision were placed in fixRNA buffer (Eurx, Gdańsk, Poland) and stored at 2–8 °C until use. After the fixRNA buffer was removed, the samples were divided into smaller parts, 600 μL of lysis buffer was added and the mixture was homogenised using a pellet pestle homogeniser (Sigma-Aldrich, Darmstadt, Germany). Total RNA was isolated from skin homogenates.

### 2.4. RNA Isolation, Qualitative and Quantitative RNA Evaluation

Isolation of total RNA from T-regulatory cells and skin homogenates was performed using the mirVana™ miRNA Isolation Kit (Life Technologies, Carlsbad, CA, USA), which provides efficient isolation of small RNA-containing total RNA. This kit is compatible with virtually all cell and tissue types, and has been used previously to isolate RNA from body fluids and tissues [42,43,44]. Isolation of total RNA was carried out following the manufacturer’s recommendations. Qualitative and quantitative evaluation of the isolated RNA was performed spectrophotometrically by measuring the absorbance with the Eppendorf BioPhotometerTM Plus apparatus (Eppendorf, Hamburg, Germany), at 260/280 nm wavelengths. The prepared RNA was divided into portions and frozen at −80 °C for real-time polymerase chain reaction (qPCR) analysis.

### 2.5. Evaluation of Gene Expression

The evaluation of gene expression was performed analogously to the previously described procedure [44]. The reverse transcription (RT) reaction was performed using the High-Capacity cDNA Reverse Transcription Kit (Applied Biosystems, Carlsbad, CA, USA) in a volume of 20 μL. The reaction mixture contained 10 × RT buffer, 25 × dNTP Mix (100 mM), 10 × RT Random Primers, MultiScribe™ Reverse Transcriptase, RNase Inhibitor and nuclease-free water. Each sample included 100 ng of total RNA in the reaction mixture. A negative control was included using water instead of RNA. The following RT reaction conditions were used: 10 min at 25 °C, 120 min at 37 °C, 5 min at 85 °C and cooling at 4 °C.

Relative gene expression was assessed by real-time polymerase chain reaction (qPCR) using a 7900HT Fast Real-Time PCR System device (Applied Biosystems, Carlsbad, CA, USA). A total reaction volume of 20 μL contained: cDNA (1–100 ng), KAPA PROBE FAST qPCR Master Mix (2X) ABI Prism™ (Kapa Biosystems Ltd., London, UK), RNAse-free water and 20 × TaqMan^®^ Gene Expression Assay for the following genes: *IL-10* (Hs00961622_m1), *IL-17A* (Hs00174383_m1), *IL-17RA* (Hs01056316_m1), *IL-23A* (Hs00372324_m1), *IL-23R* (Hs00332759_m1) and *GAPDH* (Hs99999905_m1) selected as the reference gene in the qPCR reaction. The relative expression level of the studied genes was evaluated by the delta-delta CT method (TaqMan Relative Quantification Assay software, Applied Biosystems) and presented as the RQ value relative to the *GAPDH* reference gene. The calibrator for which RQ = 1 was RNA isolated from biological material from a healthy patient, without skin lesions. RQ > 1 indicates higher expression of the gene in test samples than in the calibrator sample, and RQ < 1 indicates less expression.

### 2.6. Dermatological Scales

The severity of skin lesions was assessed using the PASI (Psoriasis Area and Severity Index), BSA (Body Surface Area) and DLQI (Dermatology Life Quality Index) scale.

PASI is the best known and most frequently recommended measurement scale for assessing the extent and severity of psoriasis. It determines the percentage of skin affected by psoriatic lesions in four main body areas: head, trunk, upper and lower extremities. Then, within each location, three parameters are assessed, *viz.* erythema, infiltration and desquamation, on a scale from 0 (no change) to 4 (very strong intensity). The test yields a single score in the range from 0 to 72 points, with a higher PASI score indicating a greater degree of psoriatic severity and extension [45,46,47,48,49]. A PASI result below 10 points indicates mild psoriasis, and one of 10 or above that the patient has a moderate or severe form of the disease.

The BSA tool is used to determine the percentage (from 0 to 100%) of the area of the skin covered by psoriatic lesions. When calculating this scale, the Wallace rule of nines [45] is followed, in which the surface of the head represents 9% of the surface of the body, each of the upper limbs is also 9%, the front and back torso each 18% (36% in total), each lower limb 18%, and the crotch area 1%. In addition, the surface of the patient’s hand constitutes 1% of the body. Values less than 10% indicate mild psoriasis, and values greater than or equal to 10% indicate moderate to severe psoriasis. Unfortunately, unlike PASI, the BSA index does not assess morphological features of lesions, such as infiltration, erythema and scale, which means that it should not be the only ratio used in assessment.

DLQI is an easy-to-use and concise questionnaire consisting of ten questions designed for a subjective assessment of the impact of psoriasis on the patient’s quality of life in the previous week. Each question concerns the impact of this dermatological disease on a particular area of life and is rated on a scale from 0 to 3. The total number of points ranges from 0 points, indicating that psoriasis has no effect on quality of life, to 30 indicating a very strong negative impact on daily life [45,50]. The questionnaire is completed by the patient, without the help of a doctor.

The patients were divided into groups based on the questionnaire results, i.e., PASI ≤ 10 and >10, BSA ≤ 20 and >20, DLQI ≤ 10 and >10, and the relative expression of the tested cytokine genes was recorded for each of them.

### 2.7. Statistical Analysis

The results (RQ value) are shown as medians. Statistical analysis was performed using Statistica 13.1 software (StatSoft, Cracow, Poland). The Mann–Whitney U test was used to assess the statistical dependence between the study groups. Spearman’s rank correlation coefficient was used to measure the direction and strength of the relationship for individual variables. The level of correlation was fixed in the following categories: very strong (rho ≥ 0.80), moderate (rho = 0.60–0.79), fair (rho = 0.30–0.59) and poor (rho ≤ 0.29). Statistical significance was assumed for *p*-values < 0.05.

## 3. Results

### 3.1. IL-10, IL-17A, IL-17RA, IL-23A and IL-23R Gene Expression in Psoriatic Patients vs. Healthy Volunteers

The relative expression level of genes *IL-10*, *IL-17A*, *IL-17RA*, *IL-23A* and *IL-23R* was determined in the material derived from skin sections and from T-regulators lymphocytes (CD4/CD25). The median RQ values for the studied genes and the number of samples with decreased/increased expression compared to housekeeping gene values are presented in Table 1 and Table 2.

For *IL-10*, the median gene expression (RQ value) in skin was 0.078 in psoriatic patients and 0.0312 for healthy volunteers; however, in lymphocytes, it was below detection levels for both groups. In case of *IL-17A*, the median RQ in skin was 119.636 in the psoriasis group and 2.217 in the control group, but below detection levels for both groups in lymphocytes. For *IL-17RA*, the RQ values were 0.253 in psoriatic skin, 1.014 in healthy skin, 11.529 in lymphocytes from the psoriatic group and 15.458 in lymphocytes from healthy controls. For *IL-23A*, median expression was 0.231 in psoriatic skin and 0.130 for control skin, 2.893 for psoriatic lymphocytes and 6.573 for control lymphocytes. For *IL-23R*, median RQ was 46.695 in psoriatic skin and 7.018 control skin; however, the values were below detection levels in both groups of lymphocytes.

**Figure 1:**
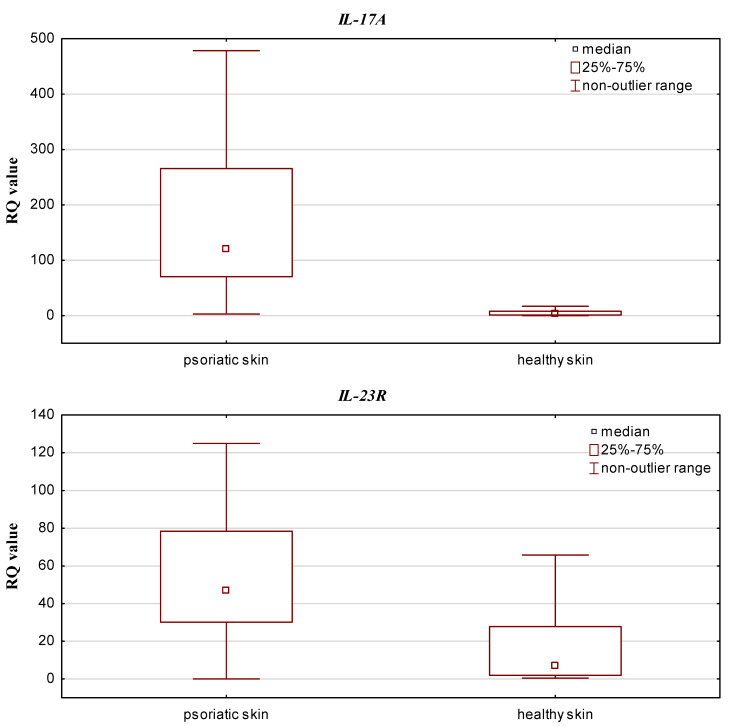
Box-and-whisker plots presenting higher expression levels (median RQ values) in psoriatic skin vs. healthy skin (controls) for *IL-17A* and *IL-23R*.

**Figure 2:**
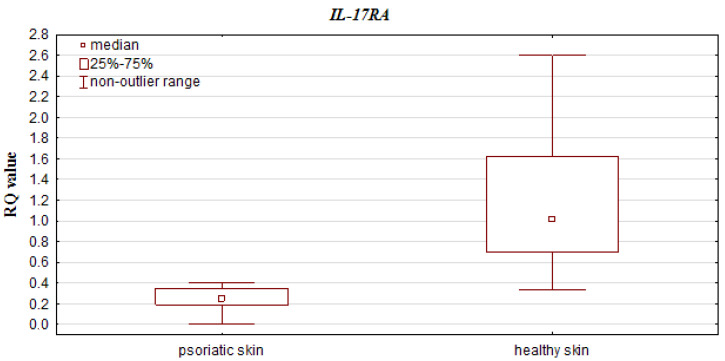
Box-and-whisker plots, presenting lower expression levels (median RQ values) in psoriatic skin vs. healthy skin (controls) for *IL-10* and *IL-17RA*.

The qPCR results (RQ values) were obtained for all psoriatic skin and control samples (healthy skin). *IL-17A* and *IL-23R* expression was upregulated in both groups compared to the calibrator. In addition, *IL-17RA* expression was decreased in psoriatic skin and elevated in the control group, while *IL-10* and *IL-23A* expression was downregulated in both groups. Statistically significant differences in relative expression level of *IL-17A* and *IL-23R* were observed between the psoriatic skin and the control group (*p* = 0.0000001 and *p* = 0.000007, respectively; Mann–Whitney U test), with higher expression observed in the psoriatic patient group (see Figure 1). Significantly lower *IL-10* and *IL-17RA* expression was found in psoriatic skin than in controls (*p* = 0.000005 and *p* = 0.0000001, respectively; Mann–Whitney U test) (Figure 2).

Regarding the biological material derived from T-reg lymphocytes (CD4/CD25), the RQ values of *IL-10*, *IL-17A* and *IL-23R* were below detection levels. The RQ values of *IL-23A* and *IL-17RA* in T-reg lymphocytes (CD4/CD25) were upregulated relative to the housekeeping gene in the psoriatic and control group. In addition, participants diagnosed with psoriasis demonstrated lower RQ values for *IL-23A* and *IL-17RA*; however, the obtained results were not statistically significant (*p* = 0.439585 and *p* = 0.254683, respectively; Mann–Whitney U test).

### 3.2. RQ Value Genes vs. Clinical Features (PASI, BSA, DLQI) of Psoriatic Patients

In the researched psoriatic group, PASI values ranged from 3.0 to 25.4 (mean 9.58), BSA from 7.5 to 61 (mean 23.83), while DLQI from 0 to 30 (mean 13.13).

The relative expression of *IL-10*, *IL-17A*, *IL-17RA*, *IL-23A* and *IL-23R* genes was analysed depending on the classification of psoriatic patients, according to the used scales (PASI, BSA, DLQI). Regarding the biological material derived from skin sections, a positive correlation was found between the RQ value of *IL-23A* and PASI (rho = 0.278984, *p* = 0.030881; Spearman’s rank correlation coefficient), and a negative correlation between the RQ value of *IL-23R* and BSA (rho = −0.265377, *p* = 0.040436; Spearman’s rank correlation coefficient). No significant correlations were found between the RQ values of the *IL-10*, *IL-17A* and *IL-17RA* genes and PASI, BSA or DLQI scores (*p* > 0.05; Spearman’s rank correlation coefficient).

**Figure 3:**
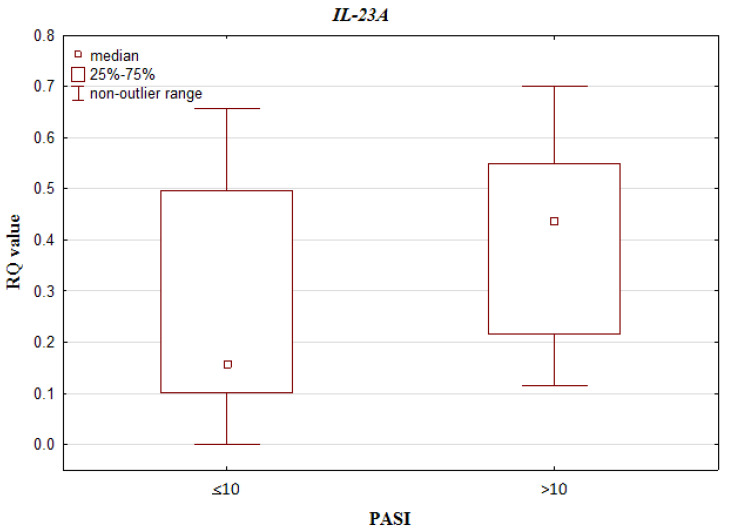
Box-and-whisker plots, presenting relative expression levels (median RQ values) of *IL-23A* in psoriatic patients with PASI ≤ 10 vs. PASI > 10.

Higher RQ values of *IL-23A* were observed in patients with PASI > 10 than PASI < 10 (0.158 vs. 0.436) (*p* = 0.010699; Mann–Whitney U test) (see Figure 3). No significant differences were observed for *IL-10*, *IL-17A*, *IL-17RA* and *IL-23R* gene RQs between patients with high and low PASI scores (*p* = 0.234864, *p* = 0.865768, *p* = 0.247344, *p* = 0.450768, respectively; Mann–Whitney U test).

**Figure 4:**
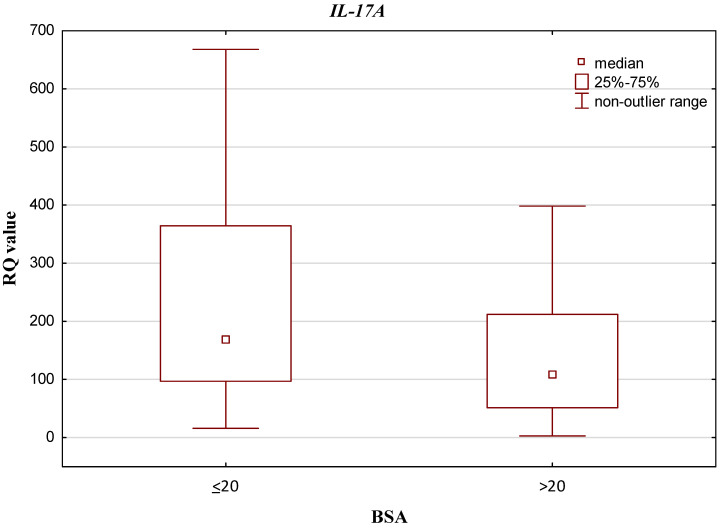
Box-and-whisker plots, presenting relative expression levels (median RQ values) of *IL-17A* in psoriatic patients with BSA ≤ 20 vs. BSA > 20.

Regarding BSA, *IL-10*, *IL-17A*, *IL-17RA*, *IL-23A* and *IL-23R* demonstrated higher RQ values among patients with BSA ≤ 20 than among those who had BSA > 20 (0.081 vs. 0.076, 168.586 vs. 108.592, 0.260 vs. 0.224, 0.234 vs. 0.231 and 51.366 vs. 36.545; respectively). Of these, only *IL-17A* demonstrated a statistically significant difference in relation to the BSA scale (*p* = 0.042520; Mann–Whitney U test). The results are presented in Figure 4. No such statistically significant differences were found for the remainder: *IL-10 p* = 0.326967, *IL-23A p* = 0.622077, *IL-17RA p* = 0.246764 and *IL-23R p* = 0.115034; Mann–Whitney U test.

Regarding DLQI, *IL-10*, *IL-17A* and *IL-17RA* demonstrated higher RQ values in the DLQI ≤ 10 group than the DLQI > 10 group (0.084 vs. 0.066, 148.100 vs. 110.145 and 0.264 vs. 0.234, respectively). In contrast, *IL-23A* and *IL-23R* demonstrated lower RQ in DLQI ≤10 than in DLQI > 10 group (0.220 vs. 0.233, 39.046 vs. 53.525, respectively). None of these differences were statistically significant with the p-values being *p* = 0.414612, *p* = 0.296663, *p* = 0.318285, *p* = 0.987968 and *p* = 0.39990 for *IL-10*, *IL-17A*, *IL-17RA*, *IL-23A* and *IL-23R*, respectively; Mann–Whitney U test.

Regarding the biological material derived from T-reg lymphocytes (CD4/CD25), no significant correlations were found regarding the expression level of *IL-10*, *IL-17A* and *IL-23R* and patient classification (PASI, BSA, DLQI) (*p* > 0.05, Mann–Whitney U test).

## 4. Discussion

Psoriasis is a disease with a complex etiopathogenesis characterized by excessive growth and aberrant differentiation of keratinocytes; however, despite much research, it remains not fully understood. It is known, however, that disorders in the immune system play a very important role. The present study examines the expression of the genes *IL-10*, *IL-17A*, *IL-17RA*, *IL-23A* and *IL-23R* in the skin and peripheral blood of patients with psoriasis and of healthy people, to identify potential factors regulating the development of psoriatic lesions. Understanding the molecular basis of this mechanism may allow the development of improved forms of tailored therapy.

### 4.1. IL-10

*IL-10* is a homodimeric anti-inflammatory protein, which acts as an immunoregulator which inhibits the synthesis of other cytokines. It is encoded by the *IL-10* gene. It is produced mainly by monocytes, stimulated T lymphocytes (especially Th2, but also Treg) and B lymphocytes, macrophages and keratinocytes. *IL-10* is believed to inhibit the inflammatory response and regulate cell-mediated immune processes by inhibiting the synthesis of INF-γ by Th1 lymphocytes and *IL-1α*, *IL-6*, *IL-8*, *IL-12*, TNF-α, G-CSF and GM-CSF by monocytes or macrophages. In addition, it can suppress the ability of antigen-presenting cells (mainly monocytes) and stimulate the production of IL-1 receptor antagonists [51,52,53,54,55].

Our findings indicate a relative deficiency in cutaneous *IL-10* mRNA expression in both study groups, but that levels were significantly lower in patients with psoriasis. This is consistent with Cheng et al. [56], who report a relative deficiency in *IL-10* mRNA expression in psoriatic lesions compared with normal skin tissues. Similar relationships were found in the work of Asadullah et al. [51], who observed a low level of *IL-10* expression in the psoriatic skin versus skin with other inflammatory diseases. This is also supported by immunohistochemical results suggesting a low cutaneous *IL-10* protein expression in psoriasis [57].

However, conflicting data have also been published—Wolk et al. report elevated expression of *IL-10* in biopsies from patients with psoriasis relative to healthy donors [58], but Uyemura et al. [59] and Schlaak et al. [60] weakly or not at all detected *IL-10* mRNA. In addition, exogenous supply of *IL-10* in treatment has been found to increase intradermal *IL-10* mRNA expression [51] and significantly decrease PASI [61]. The clinical effectiveness of *IL-10* application has also been confirmed by Reich et al. [62] and McInnes et al. [63].

This suggests that *IL-10* may have anti-psoriatic effects and could be an alternative form of therapy for psoriasis. Despite this, a randomised, placebo-controlled, double-blind 12 week treatment trial of recombinant human *IL-10* failed to markedly change baseline PASI score [64]. This is confirmed by the fact that not all laboratory tests analysing the pathological processes underlying the development of the disease result in the subsequent registration of the medical product. The results of laboratory observations have been verified by drug studies on the safety and effectiveness of therapy.

The RQ of *IL-10* in CD4/CD25 T-regulatory lymphocytes was below detection for both patients and healthy people, indicating that the gene is not expressed in CD4/CD25. This is also confirmed by the results of researchers from the Berlin Humboldt University [51], which indicate that *IL-10* mRNA expression was not elevated in peripheral blood monoclonal cells from psoriatic patients in comparison to controls. In addition, the same group of scientists observed that *IL-10* mRNA expression in peripheral blood mononuclear cells was higher during conventional anti-psoriatic therapy than before treatment. This may suggest that *IL-10* has an anti-psoriatic effect.

Although no significant relationships were observed between the level of *IL-10* expression and the PASI, BSA or DLQI value in all patients, higher *IL-10* expression was observed in the BSA ≤ 20 and DLQI ≤ 10 groups. It has previously been found that therapy with the use of recombinant *IL-10* did not significantly decrease PASI value [64], suggesting that cutaneous levels of *IL-10* are not indicative of disease severity. In contrast, many studies show a reduction in PASI score after the *IL-10* treatment, but no total recovery [51,52,61,62,63,65].

It is possible therefore that *IL-10* might have a certain role in the regression of psoriasis and may be considered as a therapeutic approach; however, further investigations are needed to establish its exact anti-psoriatic role.

### 4.2. IL-17A and IL-17RA

The interleukin 17 family includes six members labelled from A to F [66,67]. Although all are produced by T-helper lymphocytes, particularly *IL-17A*, in response to stimulation by various compounds, including *IL-23* [68,69,70], *IL-17A* is also produced in small amounts by other cells such as natural killer (NK) cells, neutrophils and mastocytes. Biologically active *IL-17A* binds to the receptor *IL-17RA*, resulting in the activation of signalling cascades (including increased production of cytokines, such as TNF-α, *IL-6*, transforming growth factor (TGF)-β, IL-1β, G-CSF and GM-CSF), which in turn provoke the induction of chemokines that attract monocytes, neutrophils and dendritic cells to the place of inflammation [71]. Such an imbalance in the level of cytokines and chemokines allows uninhibited proliferation of Th17 lymphocytes and the production of *IL-17* in psoriatic lesions; this plays a key role in the pathogenesis of this disease, because *IL-17A* is a factor leading to the dysfunction of epidermal keratinocytes and promotes their abnormal differentiation [72].

*IL-17RA* is a ubiquitous glycoprotein associated with the biological membrane structure, and is expressed in many tissues, including epithelial cells in abundance [71]. It is one of five receptors for *IL-17* family [73,74]. It interacts with both *IL-17A* and *IL-17F*, but a heterodimeric junction consisting of both *IL-17RA* and also *IL-17RC* is required to transmit the signal for these interleukins [66,73].

Li et al. [75], Teunissen et al. [76], Zaba et al. [77], Lowes et al. [78] and Haider et al. [79] reported significantly higher expression of *IL-17* mRNA in psoriatic lesions than in healthy controls. Chan et al. [80] described the same relationship for *IL-17A* mRNA expression. This is in line with the results of the present study. Similarly, previous studies have noted greater expression of mRNA encoding *IL-17A* or *IL-17* in lesional than non-lesional biopsies of patients with psoriasis [81,82]. These findings suggest that up-regulated *IL-17* expression may be related to the pathogenesis of psoriasis by amplifying the development or sustaining chronic inflammatory responses in the skin. This has been confirmed by the effectiveness of anti-*IL-17* biological drugs [83,84,85,86].

Our present results show decreased expression of *IL-17RA* in psoriatic skin and an increase in healthy individuals. In contrast, Johansen and colleagues [81] reported no difference in the *IL-17RA* mRNA expression level between lesional and non-lesional psoriatic skin; however, it should be noted that the study group only included nine patients. It may be possible that no significant relationship exists between the mRNA levels of the *IL-17R* family members and their corresponding ligands [81], or that cellular responses to the *IL-17A* (and *IL-17F*) stimulation require expression of the entire *IL-17RA* and *IL-17RC* receptor complex, not just *IL-17RA* alone [66,73,87,88,89].

Our present findings indicate that *IL-17* gene expression was below detection levels in CD4/CD25 lymphocytes in both patient and control groups. However, Suárez-Fariñas et al. [82] reported increased expression of *IL-17* in the blood of psoriasis patients compared with healthy population. Interestingly, in the present study, *IL-17A* receptor gene expression was elevated in peripheral blood lymphocytes in both study groups. The presence of *IL-17* expression in the skin but not on CD4/CD25 blood cells may indicate local rather than systemic involvement in the inflammation process in psoriasis.

In addition, no significant correlation was found between the level of *IL-17A* and *IL-17RA* expression and the PASI, BSA or DLQI value in any patient. However, the group with mild psoriasis (BSA ≤ 20 and DLQI ≤ 10) demonstrated higher levels of expression of *IL-17A* and *IL-17RA*. These findings are consistent with those of Hijnen et al. [90], who also reported that level of expression of *IL-17*, but also *IL-22*, *IFN-ɤ* and *IL-13*, in skin biopsies of patients with psoriasis did not correlate with PASI. This may be explained by the argument that the local cytokine production in single plaque does not reflect the general activity of the disease. Previous studies report a positive correlation between *IL-17A* and psoriasis severity, but they assessed *IL-17A* protein level in serum or tissue, not gene expression [91,92,93,94,95]. In support of this hypothesis, recent data indicate that anti-*IL-17* agents, such as secukinumab (anti-*IL-17A* antibody), ixekizumab (anti-*IL-17A* antibody), brodalumab (anti-*IL-17RA* antibody) and bimekizumab (anti-*IL-17A* and -17F antibody) are effective forms of treating moderate to severe psoriasis, which is expressed by reducing PASI and/or BSA score [83,84,85,86,96,97,98,99,100,101,102,103].

*IL-17A* seems to fulfil a key role in the pathway of the pathogenesis of psoriasis, and biological therapies targeting *IL-17A* and/or *IL-17RA* yield remarkable effects for inflammatory skin dermatoses like psoriasis.

### 4.3. IL-23A and IL-23R

*IL-23* is a heterodimer cytokine consisting of two subunits. The first, marked as p19 (subunit A, *IL-23A*), is specific, homologous to the p35 subunit of *IL-12*, and encoded by the *IL-23A* gene. The second subunit is p40 (subunit B, *IL-12*B), common to *IL-23* and *IL-12*, encoded by the *IL-12B* gene [71]. Activated dendritic cells, macrophages or monocytes and keratinocytes are the main sources of *IL-23* [104,105,106,107]. The receptor for *IL-23* is a complex consisting of two elements: *IL-12Rβ1* (*IL-12RB1*), which binds to the p40 subunit, and *IL-23R*, which binds to the p19 subunit [71,108].

*IL-23* plays an important role in the proliferation, maturation and differentiation of T cells into Th1, Th17 and Th22 cells, and enables the release of *IL-6*, *IL-17*, *IL-21*, *IL-22* and GM-CSF by Th17 cells [71]. Moreover, it strongly induces the secretion of INF-γ from T lymphocytes and the production of acute-phase proteins. The whole process leads to excessive, accelerated proliferation of keratinocytes and inflammation.

In the present study, *IL-23A* gene expression was decreased in the psoriasis group and the controls. This may be because the expression of *p40* subunit, which is shared by *IL-23* and *IL-12*, can be a key factor in the development and maintenance of psoriatic skin lesions: previous studies report elevated level of gene expression of *IL-12B* in diseased skin [44,109,110]. Different results were presented by Fitch et al. [71], Chan et al. [80], Lee et al. [108] and Chamian et al. [111], who reported an increase in *p19* mRNA in psoriatic skin compared with healthy skin, as well as an elevated level of *p40* in lesional tissues, but not *p35*, which suggests *IL-23* plays a greater role in the pathogenesis of psoriasis than *IL-12*. A similar relationship with *p40* and *p35* levels was found by Cheng et al. [56]. Because the p40 subunit of *IL-12* is shared with *IL-23*, it is possible that *p40* overexpression may be incorrectly attributed to *IL-12* instead of *IL-23*. Increased levels of *IL-23* expression and *IL-12 p40* in tissue of patients with psoriasis was also observed by Tonel et al. [112]; however, they also described an elevated level of *IL-12 p35*. This is consistent with many previous studies [111,113,114,115,116], in which the level of *IL-23* (assessed by either mRNA or protein) decreased together with clinical efficacy after psoriatic treatment. Furthermore, therapy based on anti-p40 monoclonal antibody, which target both *IL-12* and *IL-23*, resulted in clinical improvement in patients with psoriasis [113,117,118,119,120].

In the case of *IL-23R*, Tonel et al. [112] reported that *IL-23* receptor expression was increased in the skin of patients with psoriasis. Similar results were obtained in the present study: while *IL-23R* expression was elevated in skin biopsies from both the patients and control groups, the increase was markedly higher for the psoriatic group.

While *IL-23A* expression was detected in T-regs (CD4/CD25) in both the patient and control groups, *IL-23R* expression was beneath the level of detection. While Kagami et al. [121] reported elevated *IL-23R* expression in peripheral blood of patients with versus without psoriasis, Tonel et al. [112] reported no such significant difference in various cells.

Our present findings indicate a positive correlation between PASI and *IL-23A* expression, i.e., the higher *IL-23* gene expression was observed in the higher PASI (>10) group. In addition, the *IL-23A* expression was significantly higher in patients with moderate to severe disease (with PASI > 10) than those with the mild (PASI ≤ 10) form of psoriasis. These results are reflected in many reports of treatment with biological drugs that affect *IL-23*: ustekinumab and briakinumab (monoclonal antibodies against the *IL-12*/*IL-23*p40 subunit), guselkumab (fully human, monoclonal *IL-23* antagonist targeting the unique p19 subunit of *IL-23*), tildrakizumab and risankizumab (humanized monoclonal antibodies inhibiting selectively the p19 subunit of *IL-23*) were found to significantly reduce PASI and BSA score [113,117,118,119,120,122]. However, in the present study, *IL-23* and *IL-23R* gene expression was elevated in the BSA ≤ 20 group, but the differences failed to reach statistical significance. Moreover, a decrease in *IL-23R* expression was observed as the BSA increased; this may be due to the fact that the BSA scale assesses only the extent of psoriasis, but not its severity, as it does not take into account the morphological features (infiltration, scale and erythema) of lesions [45,46,47,49].

Therefore, *IL-23* antagonists appear to have a therapeutic effect on psoriasis, and this selective blockade of *IL-23* subunit may have greater efficacy than the blockade of the p40 subunit, common to *IL-12* and *IL-23*. This may be because *IL-12* is a crucial cytokine involved in promotion of Th1 lymphocyte differentiation. Nevertheless, further studies comparing the roles of *IL-12* or *IL-23* in the pathogenesis of psoriasis should be conducted.

## 5. Conclusions

The exact course of the pathogenetic cascade in psoriasis is still not fully understood. Nevertheless, it is generally accepted that immune system cell mediators are important factors in the development and maintenance of psoriatic lesions. Our findings, i.e., the increased level of *IL-17A* and *IL-23R* expression, the decreased *IL-10* expression in the skin of psoriasis patients and the positive correlation between *IL-23A* and PASI index, suggest that *IL-17*, *IL-23* and *IL-10* may be important factors in the pathogenesis of psoriasis and could be valuable therapeutic targets. Only weak associations, if any, were observed between *IL-17A*, *IL-17RA* and *IL-10* expression and severity of psoriatic lesions, represented by PASI and BSA; this may result from the fact that these indicators include an assessment of the extent of the disease—a parameter that is also influenced by factors other than gene expression level. However, further studies are needed to demonstrate specific involvement of selected cytokines in the pathogenesis of psoriasis.

## Figures and Tables

**Table 1:** Gene expression levels (RQ values) in skin assessed by the ΔΔCT method and percentage of samples with expression levels above and below calibrator values in the psoriatic and control groups.

RQ Skin				
Gene		Median	RQ < 1 [n (%)]	RQ > 1 [n (%)]
*IL-10*	psoriatic	0.078	59 (98)	1 (2)
	healthy	0.312	20 (83)	4 (17)
*IL-17A*	psoriatic	119.636	0 (0)	60 (100)
	healthy	2.217	6 (25)	18 (75)
*IL-17RA*	psoriatic	0.253	56 (93)	4 (7)
	healthy	1.014	12 (50)	12 (50)
*IL-23A*	psoriatic	0.231	55 (92)	5 (8)
	healthy	0.130	18 (75)	6 (25)
*IL-23R*	psoriatic	46.695	1 (2)	59 (98)
	healthy	7.018	1 (4)	23 (96)

**Table 2:** Gene expression levels (RQ values) in lymphocytes assessed by the ΔΔCT method and percentage of samples with expression levels above and below calibrator values in the psoriatic and control groups.

RQ Lymphocytes (CD4/CD25)				
Gene		Median	RQ < 1 [n (%)]	RQ > 1 [n (%)]
*IL-10*	psoriatic	under detection level		
	healthy			
*IL-17A*	psoriatic	under detection level		
	healthy			
*IL-17RA*	psoriatic	11.529	1 (2)	59 (98)
	healthy	15.458	3 (12.5)	21 (87.5)
*IL-23A*	psoriatic	2.893	13 (22)	47 (78)
	healthy	6.573	5 (21)	19 (79)
*IL-23R*	psoriatic	under detection level		
	healthy			

## Data Availability

The data used to support the findings of this study are available from the corresponding author upon request.
